# Differential roles of human Dicer-binding proteins TRBP and PACT in small RNA processing

**DOI:** 10.1093/nar/gkt361

**Published:** 2013-05-09

**Authors:** Ho Young Lee, Kaihong Zhou, Alison Marie Smith, Cameron L. Noland, Jennifer A. Doudna

**Affiliations:** ^1^Department of Molecular and Cell Biology, University of California, Berkeley, CA 94720, USA, ^2^Howard Hughes Medical Institute, University of California, Berkeley, CA 94720, USA, ^3^Department of Chemistry, University of California, Berkeley, CA 94720, USA and ^4^Physical Biosciences Division, Lawrence Berkeley National Laboratory, Berkeley, CA 94720, USA

## Abstract

During RNA interference and related gene regulatory pathways, the endonuclease Dicer cleaves precursor RNA molecules to produce microRNAs (miRNAs) and short interfering RNAs (siRNAs). Human cells encode a single Dicer enzyme that can associate with two different double-stranded RNA (dsRNA)-binding proteins, protein activator of PKR (PACT) and trans-activation response RNA-binding protein (TRBP). However, the functional redundancy or differentiation of PACT and TRBP in miRNA and siRNA biogenesis is not well understood. Using a reconstituted system, we show here that PACT and TRBP have distinct effects on Dicer-mediated dsRNA processing. In particular, we found that PACT in complex with Dicer inhibits the processing of pre-siRNA substrates when compared with Dicer and a Dicer–TRBP complex. In addition, PACT and TRBP show non-redundant effects on the production of different-sized miRNAs (isomiRs), which in turn alter target-binding specificities. Experiments using chimeric versions of PACT and TRBP suggest that the two N-terminal RNA-binding domains of each protein confer the observed differences in dsRNA substrate recognition and processing behavior of Dicer–dsRNA-binding protein complexes. These results support the conclusion that in humans, Dicer-associated dsRNA-binding proteins are important regulatory factors that contribute both substrate and cleavage specificity during miRNA and siRNA production.

## INTRODUCTION

MicroRNAs (miRNAs) and small interfering RNAs (siRNAs) are 21–24 nucleotide (nt) non-coding sequences that regulate gene expression by targeting mRNAs. During miRNA biogenesis, primary RNA transcripts in the nucleus are cleaved by the Microprocessor complex to produce pre-miRNAs that are exported to the cytoplasm. The endoribonuclease Dicer then catalyzes further cleavage of these pre-miRNAs to produce mature miRNAs, which regulate the translation or degradation of specific mRNAs. Dicer can also generate short interfering RNAs (siRNAs) by cleaving long double-stranded RNA (dsRNA) precursors (1,2).

Immunoprecipitation and reconstitution experiments in various systems have shown that Dicer associates with proteins in the Argonaute (Ago) family of endonucleases and with specific double-stranded RNA-binding proteins (dsRBPs) (3–7). In *Drosophila,* which encodes two distinct Dicer isoforms, the Dicer-1 protein binds to the dsRBP Loquacious (Loqs-PA, PB), which is required for efficient pre-miRNA cleavage during miRNA biogenesis. Dicer-2, the other Dicer isoform, binds to Loqs-PD and R2D2, which contribute to efficient pre-siRNA processing and siRNA loading onto Ago2 during siRNA biogenesis, respectively (8–12). In organisms including worms and humans, however, a single Dicer isoform processes both pre-miRNAs and pre-siRNAs in association with distinct dsRBP partners. In *Caenorhabditis elegans*, RDE-4 is the dsRBP associated to DCR-1 (2,6,7). In humans, these dsRBPs are protein activator of PKR (PACT) (5) and trans-activation response RNA-binding protein (TRBP) (3,4).

In association with Dicer, TRBP contributes to RNA substrate binding, product length determination and the assembly of RNA-induced silencing complexes (RISCs) (13–15). However, little is known about the function of PACT, or the underlying mechanisms by which these dsRBPs influence Dicer-mediated RNA processing. Here we used an *in vitro* reconstituted system to investigate the roles of dsRBPs in different dsRNA substrate processing reactions. The results of these experiments show that despite their similar size and three-domain architecture, TRBP and PACT affect Dicer-catalyzed substrate processing differently. TRBP and PACT have distinct activities both in determining cleavage sites to produce different sized miRNAs (isomiRs) and in distinguishing between pre-miRNA and pre-siRNA substrates for Dicer. In particular, PACT containing Dicer complexes disfavor pre-siRNA substrates compared with pre-miRNA substrates much stronger than TRBP containing Dicer complexes. Using multiple hybrid forms of TRBP-PACT proteins swapping and deleting domains and/or linker region, we found that the two N-terminal dsRNA-binding domains of TRBP and PACT confer these differential behaviors of Dicer–dsRBP complexes. These results suggest a model in which TRBP and PACT function as key regulators of miRNA and siRNA biogenesis by Dicer in substrate recognition, binding, processing and RISC loading.

## MATERIALS AND METHODS

### Expression and purification of proteins and complexes

Dicer, TRBP, Dicer–TRBP and Dicer–TRBP–Ago2 complex were prepared as reported before (15). PACT, Dicer–PACT and Dicer–PACT–Ago2 were prepared using the same methods as the complexes containing TRBP, except a 5 ml HiTrap-SP column was used after TEV cleavage to remove His_6_-MBP instead of a Ni-nitrilotriacetic acid column. The bound PACT protein was eluted from the HiTrap-SP column at 350–400 mM KCl during a linear gradient run from 150 mM KCl to 1 M KCl and subsequently loaded onto a Superdex-200 gel filtration column. Chimeric proteins P12T3 and TRBP-PL were expressed and purified using the same methods as the TRBP purification, and PACT-TL was expressed and purified using same methods as the PACT purification. For the T12P3 purification, a 5 ml HiTrap-Q column was used before the HiTrap-SP column, and the unbound flow-through fraction was loaded to 5 ml HiTrap-SP column for the next steps, which were same as the PACT purification method. Chimeric proteins were designed as follows: T12P3 has the combined sequence of TRBP residues 1–232 and PACT residues 197–313. P12T3 has the combined sequence of PACT residues 1–196 and TRBP residues 221–366. TRBP-PL has the combined sequence of TRBP residues 1–96 and 159–366, connected by PACT linker residues 97–125. PACT-TL has the combined sequence of PACT residues 1–96 and 126–313, connected by TRBP linker residues 93–158. The sequences of the chimeric proteins are available in the Supporting Information.

### RNA substrate preparation

Pre-miRNA substrates were prepared by *in vitro* transcription using T7 RNA polymerase, and the transcribed RNAs contained one ribozyme at each end for homogeneous RNA production. After *in vitro* transcription, RNAs were gel purified and end-labeled for use in the processing assay. For 5′ end labelling, RNA was incubated with T4 polynucleotide kinase (New England Biolabs Inc.) and [γ-^32^P] ATP for 1 h at 37°C. For perfect dsRNA substrates, RNA oligos were ordered from IDT with a 5′ phosphate, and gel purified. One strand of each complementary RNA oligo pair was Calf Intestinal Alkaline Phosphatase (CIP)-treated before 5′ end labelling with [γ-^32^P] ATP. Equal amounts of each complementary RNA oligo were incubated at 65°C for 10 min and slow cooled to room temperature for annealing. The sequences of RNA substrates used in this study are as follows: pre-let-7a, 5′-UGAGGUAGUAGGUUGUAUAGUUUUAGGGUCACACCCACCACUGGGAGAUAACUAUACAAUCUACUGUCUUACC-3′; pre-miR-200a, 5′-AUCUUACCGGACAGUGCUGGAUUUCCCAGCUUGACUCUAACACUGUCUGGUAACGAUGU-3′; pre-miR-34c, 5′-AGGCAGTGTAGTTAGCTGATTGCTAATAGTACCAATCACTAACCACACGGCCAGG-3′; dsRNA W1, 3′-UUACCGGAACCCGUUGCCAUUAAGCCCUAAUGGCUCUGGC-5′-Biotin, 5′-UGGCCUUGGGCAACGGUAAUUCGGGAUUACCGAGACCG-3′; dsRNA W2, 3′-UUAAUGGCAACGGGUUCCGGUAAGCCCUAAUGGCUCUGGC-5′-Biotin, 5′-UUACCGUUGCCCAAGGCCAUUCGGGAUUACCGAGACCG-3′.

### Kinetic assays

Labeled RNA substrates were processed with the indicated amount of Dicer or Dicer–dsRBP complexes in dicing buffer [20 mM Tris–HCl (pH 6.5), 1.5 mM MgCl_2_, 25 mM NaCl, 1 mM dithiothreitol and 1% glycerol]. Each time point sample was prepared by being quenched with 1.2 volumes of loading buffer (95% formamide, 18 mM ethylenediaminetetraacetic acid, 0.025% sodium dodecyl sulphate, 0.1% xylene cyanol and 0.1% bromophenol blue). Samples were heated at 70°C for 10 min before loading on a 12–15% denaturing 7 M urea polyacrylamide gel electrophoresis. The gels were dried and the amount of pre-miRNA and pre-siRNA substrates and miRNA and siRNA products were quantified with a phosphorimager (GE Healthcare).

## RESULTS

### Both TRBP and PACT form stable complexes with Dicer

**Figure 1.**
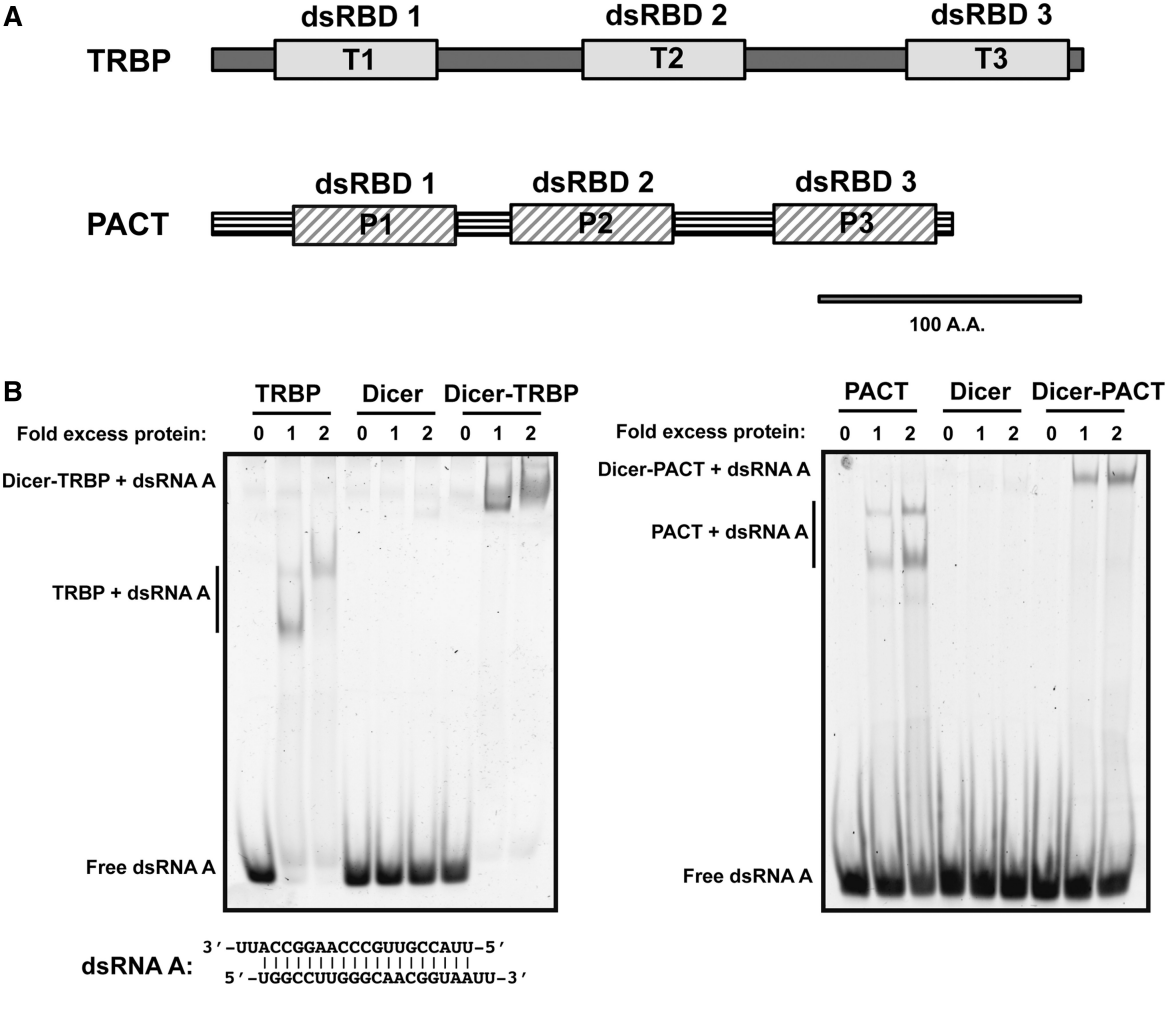
TRBP and PACT increase RNA binding of Dicer as a complex with Dicer. (**A**) Domain architectures of TRBP and PACT. Each consists of three dsRNA-binding domains. (**B**) dsRNA binding by dsRBPs and the Dicer–dsRBP heterodimers. Dicer alone does not bind to the dsRNA, but Dicer–dsRBP complexes bind to dsRNA. The gel shifts by Dicer–dsRBP complexes and by each dsRBP were distinct showing that dsRBPs help Dicer binding to dsRNA as Dicer–dsRBP complexes. dsRNA A (0.5 μM) and 0, 0.5 or 1 μM protein/complex in 20 µl of binding buffer [20 mM HEPES (pH 7.5), 300 mM KCl, 5% glycerol, 1 mM TCEP] were incubated on ice for 45 min before loading to 6% Native PAGE. The gel was stained with SYBR-Gold.

TRBP and PACT share similar architectures consisting of three dsRNA-binding domains (dsRBDs) (Figure 1A and Supplementary Figure S1), and the corresponding domains of TRBP and PACT have high sequence similarities (∼50–60% identity in Table 1). Both TRBP and PACT bind to Dicer using their C-terminal dsRBD (dsRBD3), while the two N-terminal dsRBDs (dsRBD1-2) are responsible for dsRNA binding (5,16,17). Although TRBP forms a stable Dicer–TRBP complex that has increased RNA-binding affinity relative to Dicer alone (14), the stability and consequences of PACT association with Dicer have not been clear. We tested whether PACT forms a stable complex with Dicer and also whether it changes Dicer’s affinity for dsRNA substrates. Gel filtration column chromatography and electrophoretic mobility shift assay results show that PACT associates stably with Dicer to make a Dicer–PACT complex, which increase the RNA-binding affinity of Dicer (Supplementary Figures S2–S3 and Figure1B). While both TRBP and PACT increase Dicer’s dsRNA-binding affinity, PACT has a lower affinity for dsRNA than TRBP does as the amount of dsRNA being shifted by PACT is less than that of TRBP in the same condition. Still, PACT did not dissociate from the Dicer–PACT complex as the observed RNA bands shifted by Dicer–PACT binding (Figure1B, right panel, lane 7–9) were distinct from the RNA band shifted by PACT binding (Figure1B, right panel, lane 1–3). In addition, PACT forms a stable tripartite complex with Dicer and Ago2 similar to TRBP (18).

**Table 1.** Sequence comparison between dsRBDs of TRBP and PACT

	TRBP domain 1	TRBP domain 2	TRBP domain 3
PACT domain 1	**56 (73)**	36 (42)	NS
PACT domain 2	40 (49)	**63 (79)**	NS
PACT domain 3	33 (35)	NS	**53 (75)**

The first number in the cell represents the Identity (%) and the number in parenthesis represents the Similarity/Positives (%). NS means no significant similarity. Bold values indicate high sequence similarity between corresponding domains of TRBP and PACT.

### Non-redundant effects of TRBP and PACT on Dicer processing

**Figure 2.**
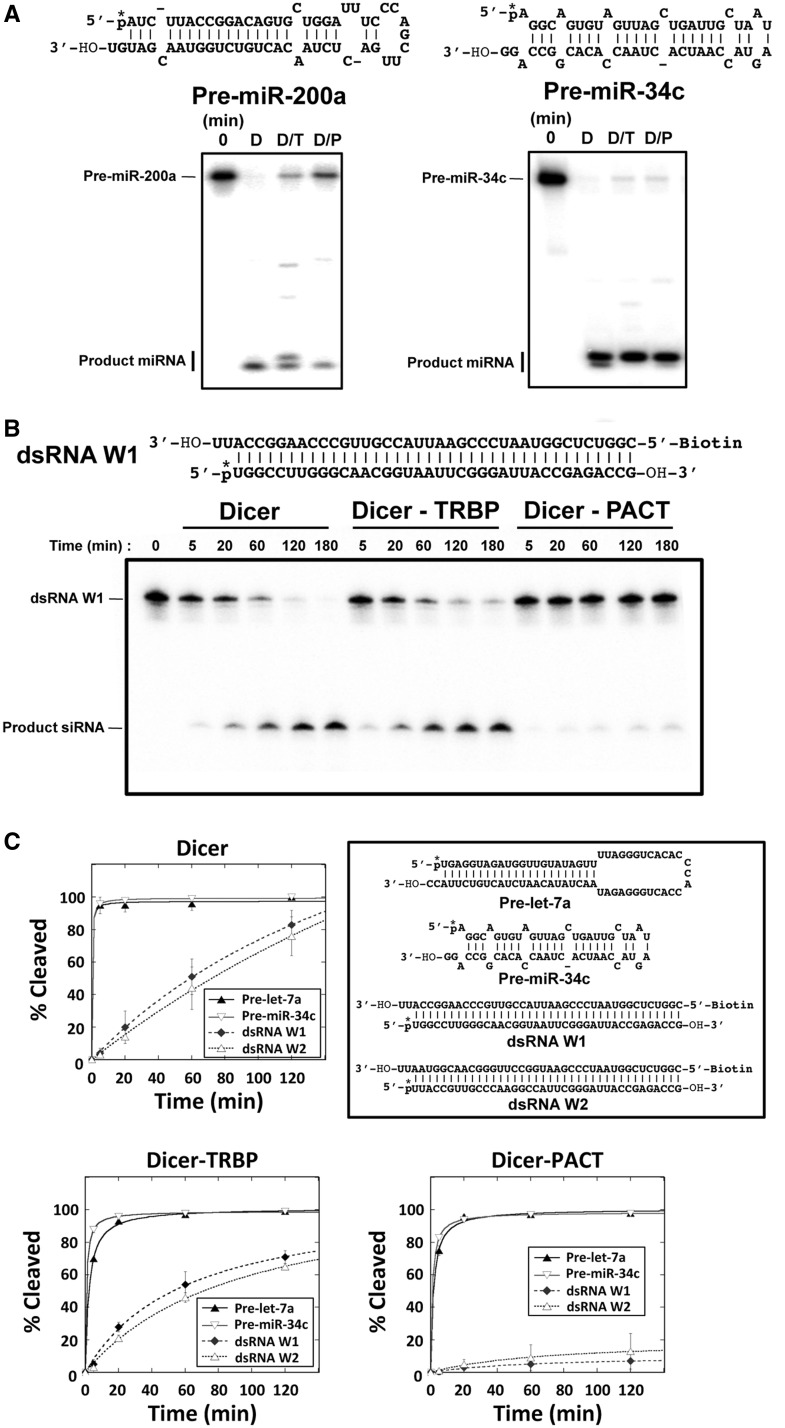
TRBP and PACT affect pre-miRNA and pre-siRNA processing by Dicer in non-redundant fashion. (**A**) Dicer–TRBP (D/T) and Dicer–PACT (D/P) are not redundant in producing different-sized miRNAs in pre-miR-200a and pre-miR-34c processing. Assays were done in a single turnover condition with 10-fold excess amount of protein/complex to RNA. RNA substrates were labeled by ^32^P phosphate at 5′ end, which is marked with an asterisk. [RNA] = 5 nM and [Dicer]/[Dicer–TRBP]/[Dicer–PACT] = 50 nM were incubated in reaction buffer for 60 min (see ‘Materials and Methods’ section) and quenched with 1.2 volumes of 2-fold formamide dye. Samples were boiled at 70°C for 10 min before loading to 12% denaturing PAGE. After drying, the gel was exposed to a phosphoscreen. (**B**) Dicer–PACT processes pre-siRNA (dsRNA W1) much less efficiently than Dicer and Dicer–TRBP in a single turnover condition with 10-fold excess amount of protein/complex to RNA. dsRNA W1 was labeled with ^32^P phosphate at 5′ end of the bottom strand, which is marked with asterisk. [RNA] = 5 nM and [Dicer]/[Dicer–TRBP]/[Dicer–PACT] = 50 nM were incubated in reaction buffer. The reactions were quenched at each time point and the following steps were the same as described in (A). (**C**) Dicer, Dicer–TRBP and Dicer–PACT processing kinetics for pre-miRNAs (pre-let-7a and pre-miR-34c) and pre-siRNAs (dsRNA W1 and dsRNA W2). Dicer–PACT processes pre-siRNA much slower than Dicer and Dicer–TRBP, while processing pre-let-7a and pre-miR-34c as efficiently as Dicer and Dicer–TRBP. A single turnover reaction condition with 10-fold excess amount of protein/complex to RNA was used for kinetic assays as in (A). The quenched samples were boiled and loaded onto 12% denaturing gel. After drying and exposing the gel to phosphoscreen, precursor substrate and product miRNA or siRNA bands were quantified by ImageQuant software (GE Healthcare life sciences). % Cleavage was calculated by 100 × (product miRNA counts)/(total counts − sum of substrate and product RNA counts).

TRBP stimulates pre-miRNA and pre-siRNA processing by increasing substrate affinity to Dicer (14). In addition, TRBP affects miRNA processing by altering both substrate specificity and product length (12,13). By contrast, the function of PACT in Dicer processing is unclear. To test the effects of PACT on Dicer-catalyzed RNA processing compared with TRBP, we tested various substrates for the processing by Dicer–dsRBP complexes, including the natural pre-miRNAs pre-let-7a, pre-miR-34c and pre-miR-200a, and synthetic pre-siRNAs, dsRNA W1 and dsRNA W2, consisting of 35 perfect base pairs with 3′ two-nucleotide single-stranded overhangs on one end (Figure 2).

The results showed that TRBP and PACT have different effects on both miRNA and siRNA processing. We found that both Dicer–TRBP and Dicer–PACT complexes process pre-miRNAs (pre-let-7a, pre-miR-34c) efficiently, but these complexes do not produce the same miRNA products in processing pre-miR-200a and pre-miR-34c (Figure 2). Dicer–TRBP produces two miRNAs and Dicer–PACT produces only one miRNA from pre-miR-200a, while Dicer–TRBP produces one miRNA and Dicer–PACT produces two miRNAs for pre-miR-34c (Figure 2A). This suggests that PACT does not affect the activity of Dicer in isomiR production at least for the two substrates tested. For pre-siRNA processing, we found that Dicer–PACT processes pre-siRNA substrates (dsRNA W1 and W2) much less efficiently than Dicer or Dicer–TRBP (Figure 2B and C). These results could not be explained by an overall low activity of Dicer–PACT as the same complex rapidly processes pre-let-7a and pre-miR-34c. Nor could these results be explained by dissociation of PACT from Dicer–PACT complex, as PACT domain 1–2 (P12), containing dsRNA-binding domains and lacking the Dicer interacting domain, shows faster pre-siRNA processing than the Dicer–PACT complex when added to Dicer processing reaction (Dicer:P12 = 1:1 ratio) (Supplementary Figures S4 and S5). This finding suggests that the inhibitory effect of Dicer–PACT in pre-siRNA processing does not result from the segregation of dsRNA substrates by free PACT or P12. The binding affinity does not explain the inhibitory effect of PACT in siRNA processing compared with Dicer either, as Dicer–PACT still binds tighter to dsRNA than does Dicer alone (Figure 1B). Furthermore, the assays were performed under single turnover conditions using a 10-fold molar excess of Dicer/Dicer–dsRBP complexes relative to RNA substrates. Thus, these results support the conclusion that Dicer–PACT has different substrate specificity compared with Dicer and Dicer–TRBP, with greatly reduced cleavage activity with pre-siRNAs, contingent on the tethering of PACT to Dicer.

### Differential effects of TRBP and PACT in complexes containing Ago2

**Figure 3.**
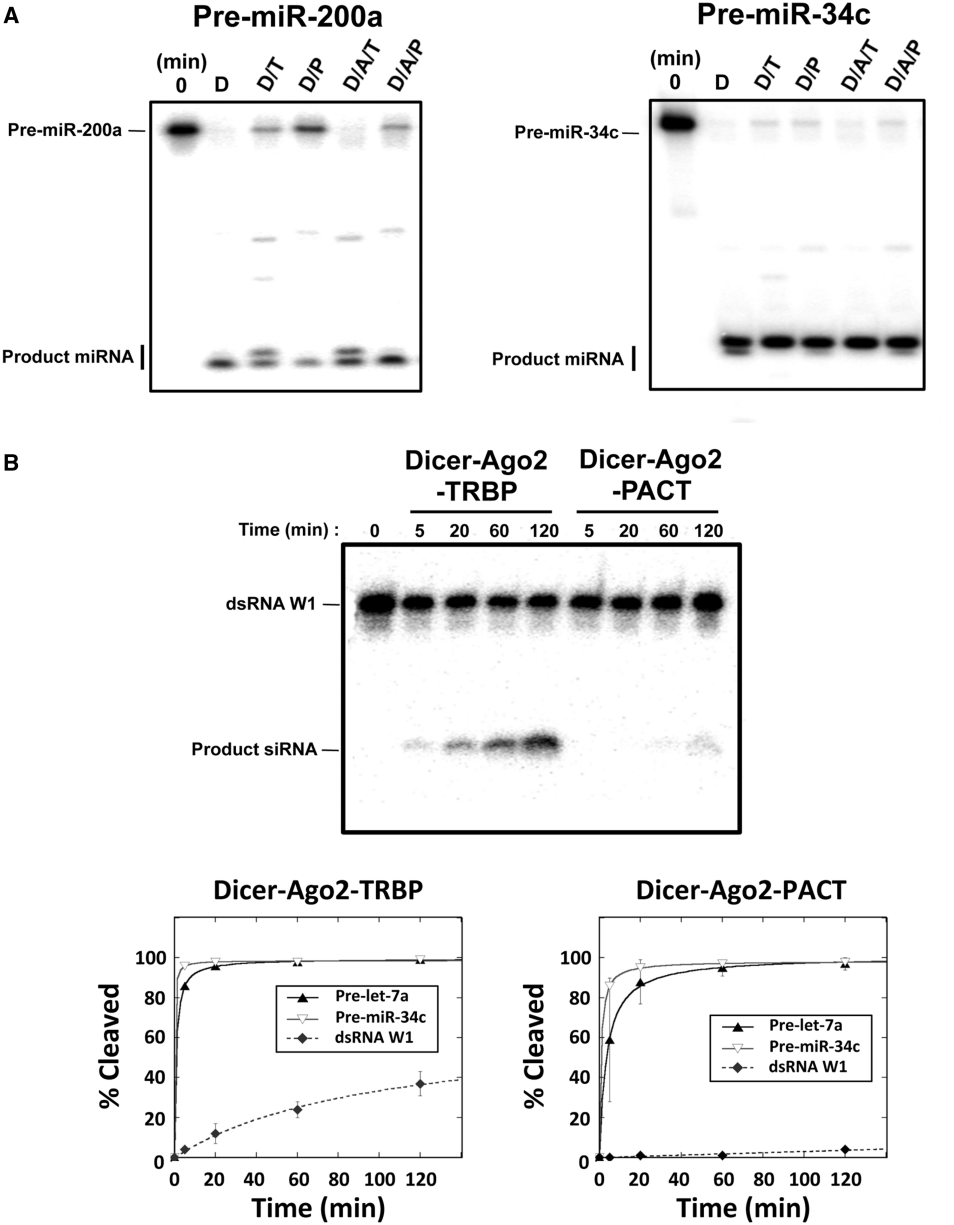
TRBP and PACT retain their effects on pre-miRNA and pre-siRNA processing by Dicer in Ago2 containing complexes: Dicer–Ago2–TRBP and Dicer–Ago2–PACT. (**A**) Dicer–Ago2–TRBP (D/A/T) and Dicer–Ago2–PACT (D/A/P) produce the same species of product miRNAs when processing pre-miR-200a and pre-miR-34c as Dicer–TRBP (D/T) and Dicer–PACT (D/P), respectively. Assays were done in a single turnover condition with 10-fold excess amount of protein/complex. [RNA] = 5 nM and [D/A/T] or [D/A/P] = 50 nM were incubated in the reaction buffer and the reaction was quenched after 60 min by adding 1.2 volume of 2-fold formamide dye. The following steps were the same as described in Figure 2A. (**B**) Dicer–Ago2–PACT (D/A/P) shows extremely low efficiency in pre-siRNA processing compared with Dicer–Ago2–TRBP (D/A/T) while processing pre-let-7a and pre-miR-34c are as fast as Dicer–Ago2–TRBP (D/A/T) as shown in Dicer–PACT and Dicer–TRBP in Figure 2B. For dsRNA W1 processing gel, each reaction has been quenched at the indicated time point and the following steps were the same as described in Figure 2B. For kinetic graphs, pre-let-7a, pre-miR-34c and dsRNA W1 were processed by Dicer–Ago2–PACT (D/A/P) and Dicer–Ago2–TRBP (D/A/T) in a single turnover condition with 10-fold excess amount of protein complex as in (A). The following steps were the same as described in Figure 2C.

In humans, TRBP, as one of the core components of RISC loading complex, helps assemble mature miRNAs or siRNAs into complexes containing Dicer, TRBP and Argonaute 2 (Ago2) and enhances the stability of the resulting assembly (3,4,15,19). As described above, we found that PACT also associates with Dicer and Ago2 to form a stable tripartite complex (18). While pre-associated Dicer–Ago2–TRBP complexes are thought to generate mature miRNAs *in vivo* (20–22), little is known about Dicer–PACT–Ago2 complexes. To test small RNA processing by such PACT-containing complexes, we tested the substrates described above for cleavage rates (pre-let-7a, pre-miR-34c and dsRNA W1) and product length specificity (pre-miR-200a and pre-miR-34c). The results of these experiments showed that Dicer–Ago2–TRBP and Dicer–Ago2–PACT produce the same miRNA products from pre-miR-200a and pre-miR-34c substrates as observed for Dicer–TRBP and Dicer–PACT, respectively (Figure 3A). In addition, both Dicer–Ago2–PACT and Dicer–Ago2–TRBP catalyze pre-let-7a and pre-miR-34c processing with similar efficiency. However, Dicer–Ago2–PACT processes pre-siRNAs with lower efficiency than Dicer–Ago2–TRBP does. These data support the conclusion that like Dicer–PACT, Dicer–Ago2–PACT also disfavors pre-siRNA processing compared with pre-miRNA processing (Figure 3B). These results demonstrate that Dicer–Ago2–TRBP and Dicer–Ago2–PACT retain the substrate specificity and product identities of Dicer–TRBP and Dicer–PACT, respectively, suggesting that dsRBPs also affect dsRNA processing in the context of Ago-containing complexes.

### N-terminal dsRNA-binding domains confer differential effects of dsRBPs on Dicer

Our results show that TRBP and PACT affect Dicer processing by altering both substrate specificity as well as product length. These differences could result from different scenarios: One possibility is that the dsRBDs of TRBP and PACT interact with RNA substrates to influence their structures and relative orientation on Dicer affecting processing rates and cleavage sites. Alternatively, the interaction between TRBP or PACT and the Dicer helicase domain could induce different conformational changes in Dicer that affect Dicer processing.

**Figure 4.**
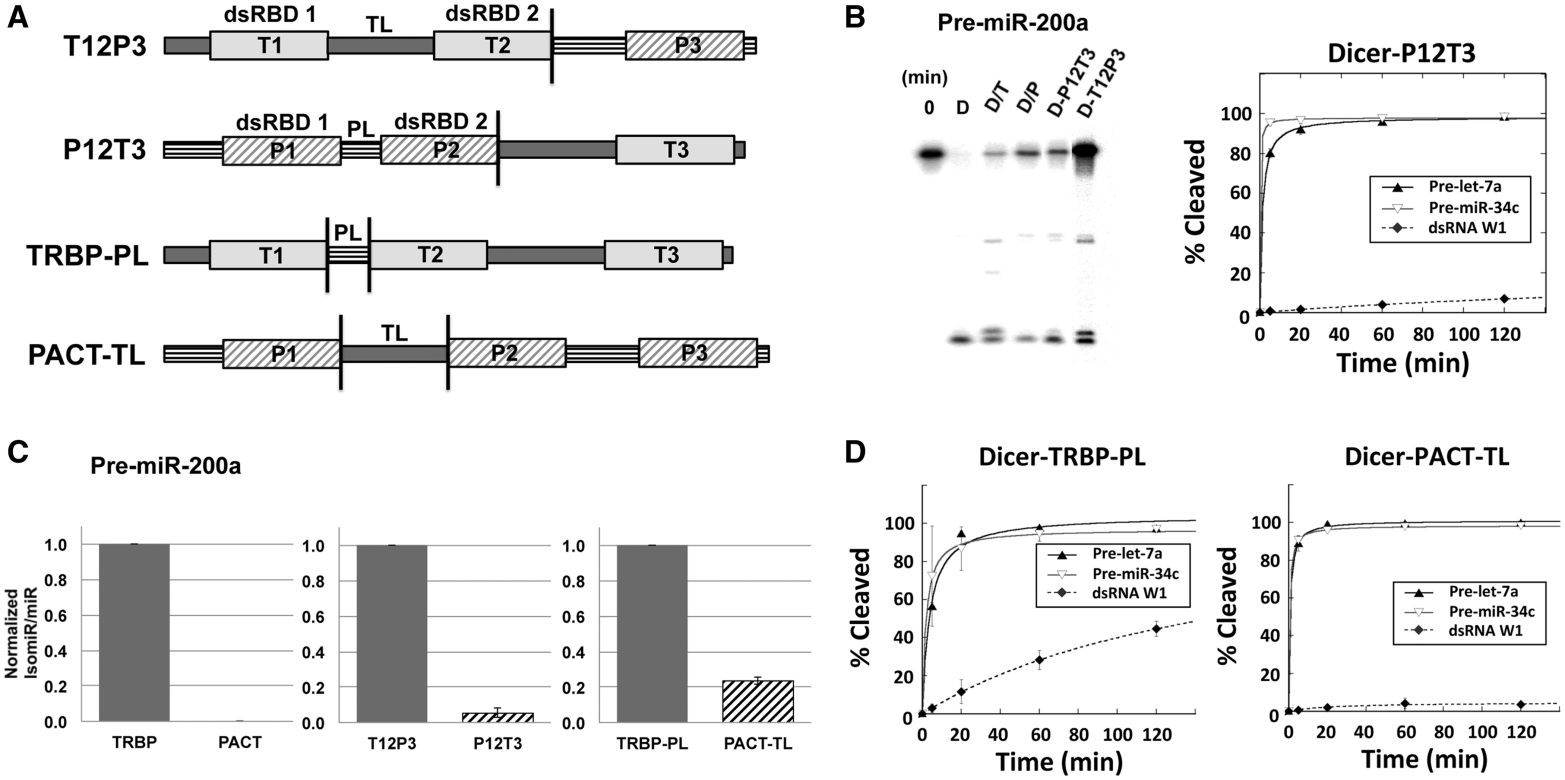
Mechanistic studies of the effects of dsRBPs on Dicer by swapping domain and linker between TRBP and PACT. (**A**) Construct design for TRBP and PACT chimeric proteins swapping the third domain (T12P3 and P12T3) and linker region between domain 1 and domain 2 (TRBP-PL and PACT-TL). (**B**) Dicer–T12P3 (D-T12P3) and Dicer–P12T3 (D-P12T3) produce highly similar product miRNAs as Dicer–TRBP (D/T) and Dicer–PACT (D/P), respectively, in processing pre-miR-200a. Dicer–P12T3 processes pre-siRNA (dsRNA W1) much less efficiently than Dicer and Dicer–TRBP as in Figure 2C, which mimics Dicer–PACT processing (Figure 2C). For pre-miR-200a processing gel, each reaction has been quenched after 60 min by adding 1.2 volume of 2-fold formamide dye. The following steps were as same as in Figures 2A and 3A. For kinetic graphs, pre-let-7a, pre-miR-34c and dsRNA W1 were processed by Dicer–P12T3 in a single turnover condition with 10-fold excess amount of protein complex. [RNA] = 5 nM and [Dicer–P12T3] = 50 nM were incubated in the reaction buffer and the reaction was quenched at each time point by adding 1.2 volume of 2-fold formamide dye. The following steps were the same as described in Figures 2C and 3B. (**C**) Dicer–TRBP-PL and Dicer–PACT-TL process pre-miR-200a producing similar isomiR products to Dicer–TRBP and Dicer–PACT, respectively. Bar graphs show relative isomiR (1nt longer miR)/miR ratio in pre-miR-200a processing by Dicer–dsRBPs (Dicer–TRBP and Dicer–PACT) and Dicer-chimeric dsRBPs (Dicer–T12P3, Dicer–P12T3, Dicer–TRBP-PL and Dicer–PACT-TL). Error bar represents Standard Deviation (SD). (**D**) Dicer–PACT-TL processes pre-siRNA (dsRNA W1) much less efficiently than Dicer–TRBP-PL, while both Dicer–PACT-TL and Dicer–TRBP-PL processes pre-let-7a and pre-miR-34c efficiently. pre-let-7a, pre-miR-34c and dsRNA W1 were processed by Dicer–PACT-TL and Dicer–TRBP-PL in a single turnover condition with 10-fold excess amount of protein complex. [RNA] = 5 nM and [Dicer–TRBP-PL]/ [Dicer–PACT-TL] = 50 nM were incubated in the reaction buffer and the reaction was quenched at each time point by adding 1.2 volume of 2-fold formamide dye. The following steps were the same as described in Figures 2C and 3B.

As mentioned earlier, both TRBP and PACT interact with Dicer using their C-terminal dsRBD3 and bind to dsRNA substrates with the two N-terminal dsRBD1-2 (5,16,17,23). To investigate the mechanism of dsRBPs’ effects on Dicer, we designed two chimeric proteins, T12P3 and P12T3, in which the third domain of TRBP and PACT has been swapped (Figure 4A). These chimeric proteins separate the two N-terminal dsRBD1-2 responsible for dsRNA binding from the third domain responsible for the interaction with Dicer so that we are able to track where the differential effect of the dsRBPs come from. These proteins were prepared as complexes with Dicer (Supplementary Figures S2 and S3) and tested for pre-miRNA and pre-siRNA processing. We found that the Dicer–T12P3 complex produces the same two miRNA products as observed for the Dicer–TRBP complex for pre-miR-200a processing (Figure 4B). Likewise, the Dicer–P12T3 complex showed results similar to those obtained with Dicer–PACT for pre-miR-200a processing producing a single miRNA product. In addition, the Dicer–P12T3 complex showed low efficiency in processing pre-siRNA dsRNA W1 (Figure 4B) similar to Dicer–PACT complex. It is noteworthy that P12T3 has the third domain from TRBP, and the interaction between Dicer and P12T3 should be as strong as the interaction between Dicer and TRBP. This result shows that the region containing two N-terminal dsRBDs of TRBP and PACT is enough to induce the effect of full-length TRBP and PACT in small RNA processing (T1-TL-T2 and P1-PL-P2, respectively in Figure 4A). In addition, there appears to be little difference in activities mediated by the C-terminal dsRBDs of TRBP and PACT in the Dicer–dsRBP complexes.

To narrow down the functional regions responsible for the differential effects of TRBP and PACT, we prepared linker-swapped proteins, TRBP-PL and PACT-TL, in which the linker region between the two N-terminal dsRBDs in TRBP and PACT (TL and PL) was switched (Figure 4A and Supplementary Figure S1). We found that Dicer–TRBP-PL produces ∼5-fold more 1-nt longer miRNA (isomiR) than Dicer–PACT-TL in pre-miR-200a processing (Figure 4C). In addition, Dicer–PACT-TL shows much less efficient pre-siRNA processing activities than Dicer–TRBP-PL, similar to Dicer–PACT and Dicer–TRBP, respectively (Figure 4D). These results suggest that the differential effects of dsRBPs on Dicer arise largely from the two N-terminal dsRBDs, dsRBD1 and dsRBD2, of TRBP and PACT.

## DISCUSSION

Double-stranded RNA-binding proteins contribute to RNAi and related pathways in eukaryotic organisms including humans, *Drosophila* and *C.elegans.* We found that the human dsRBPs TRBP and PACT, which bind directly to Dicer, alter both the kinetics and substrate specificity of Dicer-mediated RNA processing. To our surprise, PACT and TRBP work in a non-redundant fashion in small RNA biogenesis: PACT in complex with Dicer does not induce the same isomiRNA production as those observed with TRBP in pre-miRNA processing. In addition, PACT inhibits pre-siRNA processing by Dicer in Dicer–PACT complex (Figure 2) when compared with the cleavage of Dicer–TRBP and Dicer.

The differential effects of TRBP and PACT on Dicer imply that Dicer’s activity can be modulated depending on its binding partner proteins, and that TRBP and PACT are important regulatory factors for miRNA and siRNA processing. Interestingly, we observed that these differential effects of PACT and TRBP on Dicer-catalyzed RNA processing also hold true in the context of Ago2-containing complexes (Figure 3). Overall, these results suggest that these human dsRBPs play important roles in RNAi during initial substrate recognition, Dicer processing and possibly RISC loading.

While the previous publications (5,24) suggested Dicer, TRBP and PACT could associate to ternary complexes, it has been shown that either TRBP or PACT could be the dsRBP directly interacting with Dicer in Dicer–TRBP–PACT ternary complexes (24). So investigation of the functional effects of TRBP and PACT when in direct contact with Dicer would be important to understand the regulatory mechanisms of small RNA biogenesis in humans.

**Figure 5.**
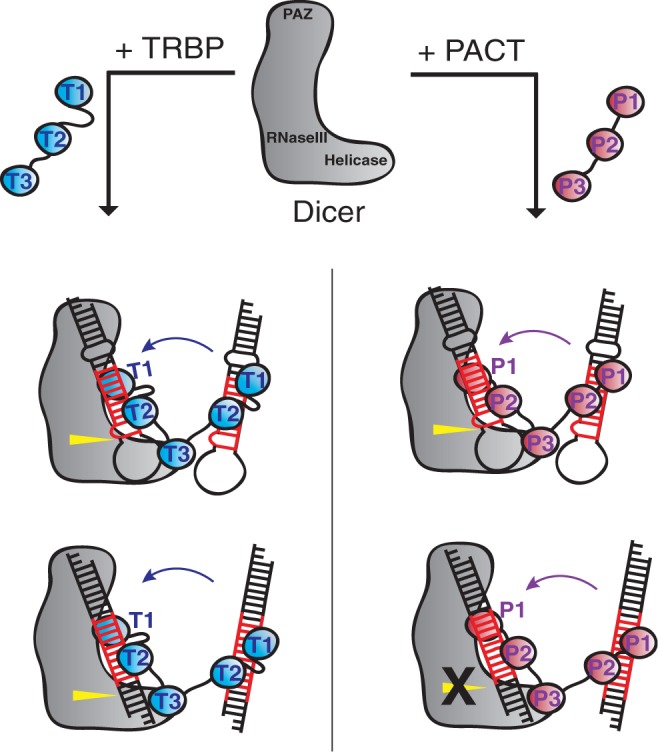
A mechanistic model showing how TRBP and PACT differentiate Dicer processing. The two N-terminal dsRBDs of TRBP and PACT bind to dsRNA substrates and recruit them to Dicer–dsRBP complexes, where cleavage occurs with dsRBPs bound to the dsRNA substrates. The two N-terminal dsRBDs of TRBP and PACT affect dsRNA structure and orientation on Dicer differently, altering Dicer processing.

Our results show that the first two N-terminal dsRBDs (dsRBD1 and 2) are the key domains for determining the differential effects of PACT and TRBP on Dicer–dsRBP complexes. Interestingly, when we tested truncated TRBP and PACT with only two N-terminal domain dsRBD1-2 (T12 and P12, respectively) with equivalent amount of Dicer for pre-miR-200a and dsRNA W1 processing, T12 and P12 were not sufficient to confer the same effects on Dicer-catalyzed RNA processing as observed for the full-length proteins (Supplementary Figure S4). As dsRBD3 is lacking, T12 and P12 do not bind directly to Dicer. This result implies that physical tethering of PACT or TRBP to Dicer influences the effects of their RNA-binding domains on RNA recruitment, relative orientation or induced conformational changes. These findings support a model in which PACT and TRBP docking on Dicer’s helicase domain recruit dsRNA substrates to Dicer and remain associated with these substrates to affect their structure and relative positioning on Dicer’s active site (Figure 5). It is possible that PACT and TRBP binding to dsRNA substrates may also influence the following steps, product release and loading of the resulting miRNAs or siRNAs into Ago2, by retaining their interaction with product dsRNAs after cleavage.

It is possible that Dicer forms complexes with either PACT or TRBP under different conditions or in different cell types, and that these complexes in turn modulate Dicer’s activity for pre-miRNA and pre-siRNA processing. The regulation of TRBP and PACT in miRNA and siRNA biogenesis could be complicated *in vivo*. The regulation of their relative expression levels and their localization has not been clear yet. In addition, there are TRBP and PACT interacting proteins such as PKR and Merlin (16,25–28) and posttranslational modifications of TRBP and PACT, which potentially alter the availability of TRBP and PACT for Dicer (19,29).

Both TRBP and PACT bind to the interferon-induced dsRNA-regulated protein kinase PKR, which binds to dsRNA and is involved in the host inflammatory response. Interestingly, TRBP is an inhibitor, whereas PACT is an activator of PKR (25–28). The interactions of TRBP and PACT with PKR are distinct from their interactions with Dicer, as the two N-terminal dsRBDs of each protein are essential for PKR binding, while the third domain is responsible for PKR regulation in each case (25). It is not known whether or how TRBP and PACT provide crosstalk between RNAi and PKR pathways. As both RNAi and PKR pathways have a role in antiviral responses, it would be interesting to understand how human dsRBPs TRBP and PACT affect RNAi and other defense pathways in normal and virus-infected cells in the future.

## SUPPLEMENTARY DATA

Supplementary Data are available at NAR Online: Supplementary Figures 1–5 and Supplementary Data Sequences.

## FUNDING

J.A.D. is a Howard Hughes Medical Institute (HHMI) Investigator. National Institutes of Health [5R01GM073794] (in part). Funding for open access charge: HHMI.

*Conflict of interest statement.* None declared.

## Supplementary Material

Supplementary Data
