# Supplementary material for: Differential roles of human Dicer-binding proteins TRBP and PACT in small RNA processing
Source: Nucleic Acids Res. 2013 May 9;41(13):6568–76. doi: 10.1093/nar/gkt361 (PMC3711433; doi:10.1093/nar/gkt361)
Supplement: Supplementary Data [file supp_41_13_6568__index.html]

Differential roles of human Dicer-binding proteins TRBP and PACT in small RNA processing — Differential roles of human Dicer-binding proteins TRBP and PACT in small RNA processing — Supplementary Data 

# Differential roles of human Dicer-binding proteins TRBP and PACT in small RNA processing

## Supplementary Data

files

**Files in this Data Supplement:**

- Supplementary Data - docx file
