# Supplementary material for: Differential roles of human Dicer-binding proteins TRBP and PACT in small RNA processing
Source: Nucleic Acids Res. 2013 May 9;41(13):6568–76. doi: 10.1093/nar/gkt361 (PMC3711433; doi:10.1093/nar/gkt361)
Supplement: Supplementary Data [file supp_gkt361_nar-00607-y-2013-File007.docx]

**Supplementary Data**

**Differential roles of human Dicer binding proteins TRBP and PACT in small RNA processing**

Ho Young Lee^1,2^, Kaihong Zhou^1,2^, Alison Marie Smith^1,2^, Cameron L. Noland^1^ and Jennifer A. Doudna^1-4,^*

Department of Molecular and Cell Biology, ^2^Howard Hughes Medical Institute and ^3^Department of Chemistry, University of California, Berkeley, CA 94720; ^4^Physical Biosciences Division, Lawrence Berkeley National Laboratory, Berkeley, CA 94720

* To whom correspondence should be addressed. Tel: 510-643-0225; Fax:510-643-0080; Email: [doudna@berkeley.edu](mailto:doudna@berkeley.edu)

Present Address: Cameron L. Noland, Genentech,Inc., South San Francisco, CA 94080, USA

**Sequences of Chimeric dsRBP proteins**

(TRBP sequence is underlined and PACT sequence is in italic)

T12P3 (TRBP 1-232 and PACT 197-313):

MSEEEQGSGTTTGCGLPSIEQMLAANPGKTPISLLQEYGTRIGKTPVYDLLKAEGQAHQPNFTFRVTVGDTSCTGQGPSKKAAKHKAAEVALKHLKGGSMLEPALEDSSSFSPLDSSLPEDIPVFTAAAAATPVPSVVLTRSPPMELQPPVSPQQSECNPVGALQELVVQKGWRLPEYTVTQESGPAHRKEFTMTCRVERFIEIGSGTSKKLAKRNAAAKMLLRVHTVPLDA

*PENHISLTNVVGHSLGCTWHSLRNSPGEKINLLKRSLLSIPNTDYIQLLSEIAKEQGFNITYLDIDELSANGQYQCLAELSTSPITVCHGSGISCGNAQSDAAHNALQYLKIIAERK*

P12T3 (PACT1-196 and TRBP 221-366):

*MSQSRHRAEAPPLEREDSGTFSLGKMITAKPGKTPIQVLHEYGMKTKNIPVYECERSDVQIHVPTFTFRVTVGDITCTGEGTSKKLAKHRAAEAAINILKANASICFAVPDPLMPDPSKQPKNQLNPIGSLQELAIHHGWRLPEYTLSQEGGPAHKREYTTICRLESFMETGKGASKKQAKRNAAEKFLAKFSNIS*MLLRVHTVPLDARDGNEVEPDDDHFSIGVGSRLDGLRNRGPGCTWDSLRNSVGEKILSLRSCSLGSLGALGPACCRVLSELSEEQAFHVSYLDIEELSLSGLCQCLVELSTQPATVCHGSATTREAARGEAARRALQYLKIMAGSK

TRBP-PL (TRBP 1-96, PACT linker 97-125 and TRBP 159-366):

MSEEEQGSGTTTGCGLPSIEQMLAANPGKTPISLLQEYGTRIGKTPVYDLLKAEGQAHQPNFTFRVTVGDTSCTGQGPSKKAAKHKAAEVAL*NILKANASICFAVPDPLMPDPSKQPKNQL*NPVGALQELVVQKGWRLPEYTVTQESGPAHRKEFTMTCRVERFIEIGSGTSKKLAKRNAAAKMLLRVHTVPLDARDGNEVEPDDDHFSIGVGSRLDGLRNRGPGCTWDSLRNSVGEKILSLRSCSLGSLGALGPACCRVLSELSEEQAFHVSYLDIEELSLSGLCQCLVELSTQPATVCHGSATTREAARGEAARRALQYLKIMAGSK

PACT-TL (PACT 1-96, TRBP linker 93-158 and PACT 126-313):

*MSQSRHRAEAPPLEREDSGTFSLGKMITAKPGKTPIQVLHEYGMKTKNIPVYECERSDVQIHVPTFTFRVTVGDITCTGEGTSKKLAKHRAAEAAI*KHLKGGSMLEPALEDSSSFSPLDSSLPEDIPVFTAAAAATPVPSVVLTRSPPMELQPPVSPQQSEC*NPIGSLQELAIHHGWRLPEYTLSQEGGPAHKREYTTICRLESFMETGKGASKKQAKRNAAEKFLAKFSNISPENHISLTNVVGHSLGCTWHSLRNSPGEKINLLKRSLLSIPNTDYIQLLSEIAKEQGFNITYLDIDELSANGQYQCLAELSTSPITVCHGSGISCGNAQSDAAHNALQYLKIIAERK*

T12 (TRBP domain 1 and 2)

MLAANPGKTPISLLQEYGTRIGKTPVYDLLKAEGQAHQPNFTFRVTVGDTSCTGQGPSKKAAKHKAAEVALKHLKGGSMLEPALEDSSSFSPLDSSLPEDIPVFTAAAAATPVPSVVLTRSPPMELQPPVSPQQSECNPVGALQELVVQKGWRLPEYTVTQESGPAHRKEFTMTCRVERFIEIGSGTSKKLAKRNAAAKMLLRVHTVPLDARD

P12 (PACT domain 1 and 2)

MSQSRHRAEAPPLEREDSGTFSLGKMITAKPGKTPIQVLHEYGMKTKNIPVYECERSDVQIHVPTFTFRVTVGDITCTGEGTSKKLAKHRAAEAAINILKANASICFAVPDPLMPDPSKQPKNQLNPIGSLQELAIHHGWRLPEYTLSQEGGPAHKREYTTICRLESFMETGKGASKKQAKRNAAEKFLAKFSN

**Supplementary Figures**

Figure S1. Sequence alignment of TRBP and PACT using NCBI blast2.

Each box indicates a dsRBD from TRBP and PACT: Blue box for dsRBD1, Red box for dsRBD2 and black box for dsRBD3.

Figure S2. PACT and chimeric dsRBPs protein purification using gel filtration column (Superdex-200). Chromatogram and protein gels are shown for each protein.


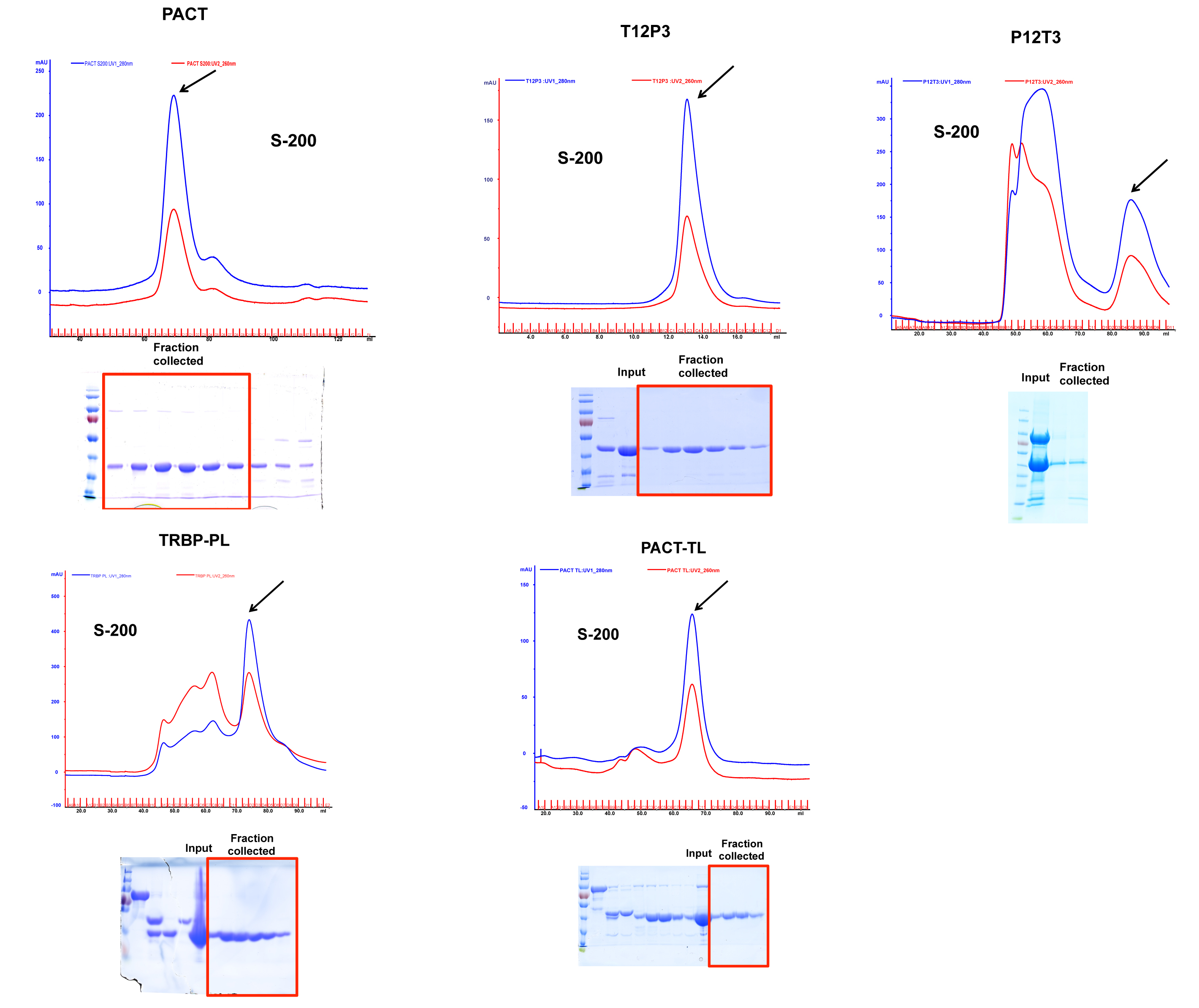


Figure S3. Dicer-PACT and Dicer-chimeric dsRBP complex formation and gel filtration purification using Superose 6 column (single or tandem Superose 6).


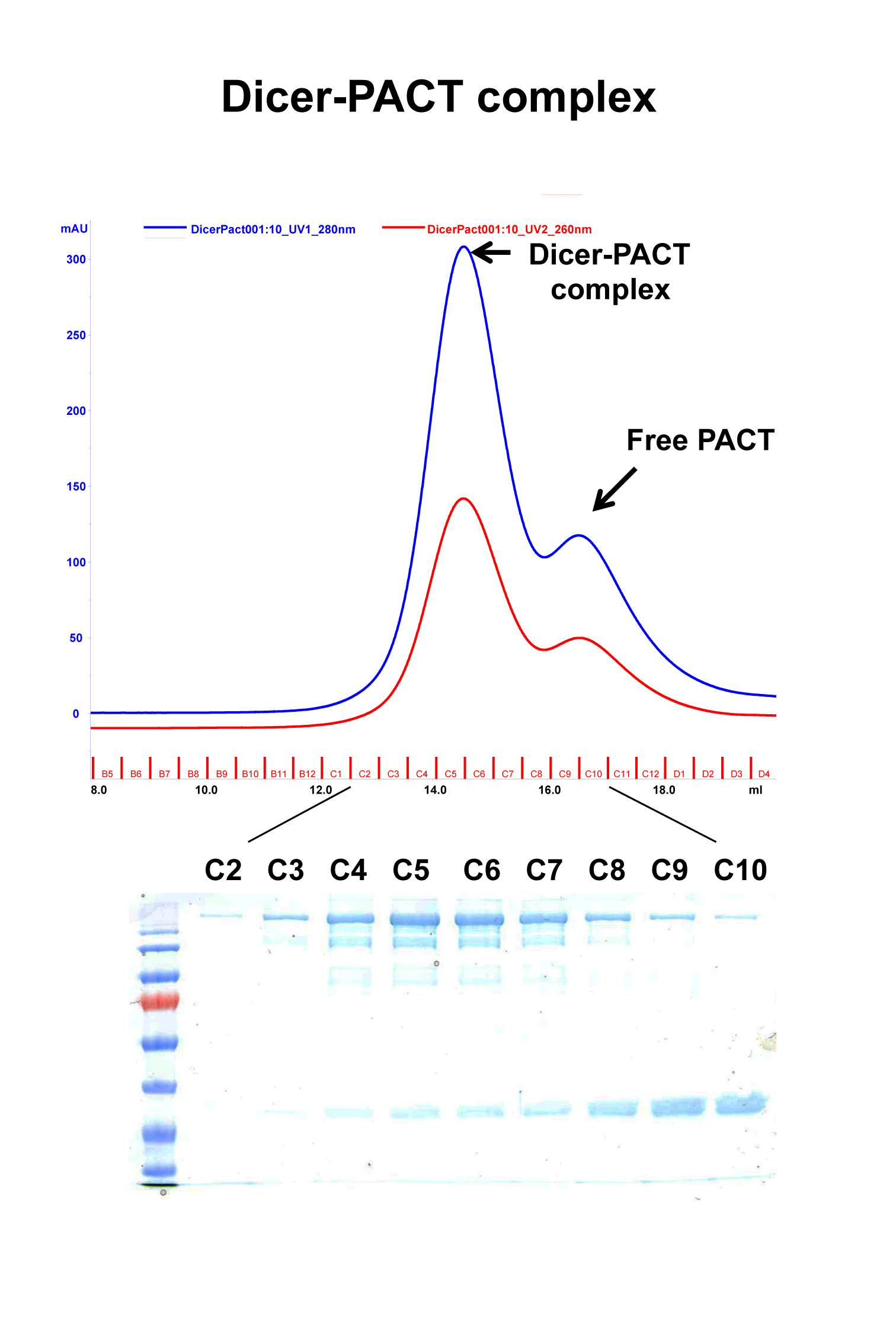


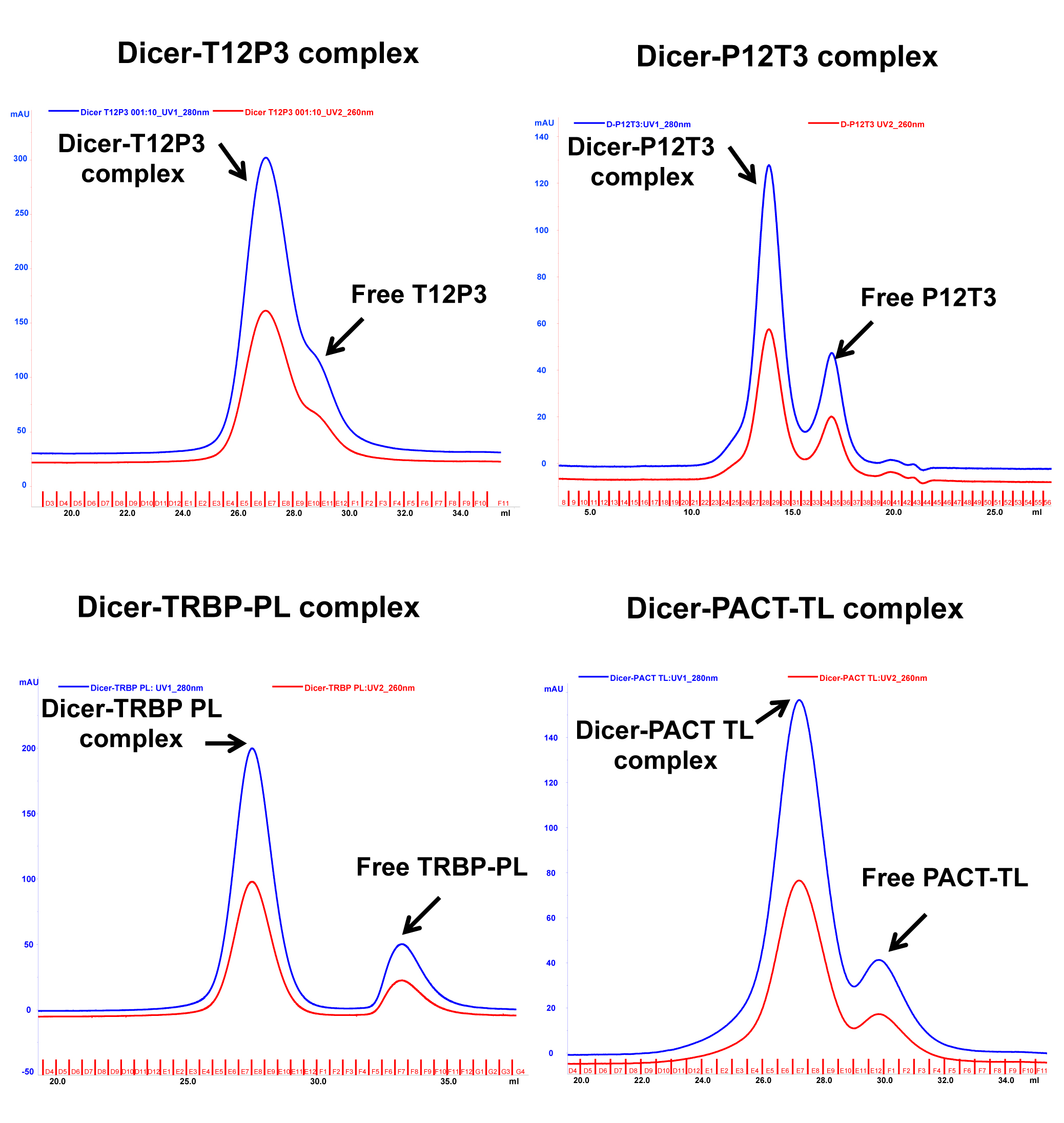


Figure S4. Truncated TRBP (TRBP dsRBD1-dsRBD2, T12) and truncated PACT (PACT dsRBD1-dsRBD2, P12) affect pre-miR-200a processing kinetics by Dicer differently from full length TRBP and PACT. For each reaction, [RNA] = 5 nM, [T12] or [P12] = 50 nM and [Dicer] = 50 nM were used in the same dicing buffer as used in Material and Methods.

Figure S5. Truncated PACT (PACT dsRBD1-dsRBD2, P12) binds pre-siRNA (37 ab dsRNA) as tightly as full-length PACT. [pre-siRNA]= 250 nM, [P12] /[PACT] = 500 nM in 20 μL buffer (20 mM Tris–HCl (pH 6.5), 1.5 mM MgCl_2_, 25 mM NaCl, 1 mM dithiothreitol and 1% glycerol) were incubated on ice for 60 min before loading to 6% Native PAGE. The gel was stained with SYBR-Gold.

_
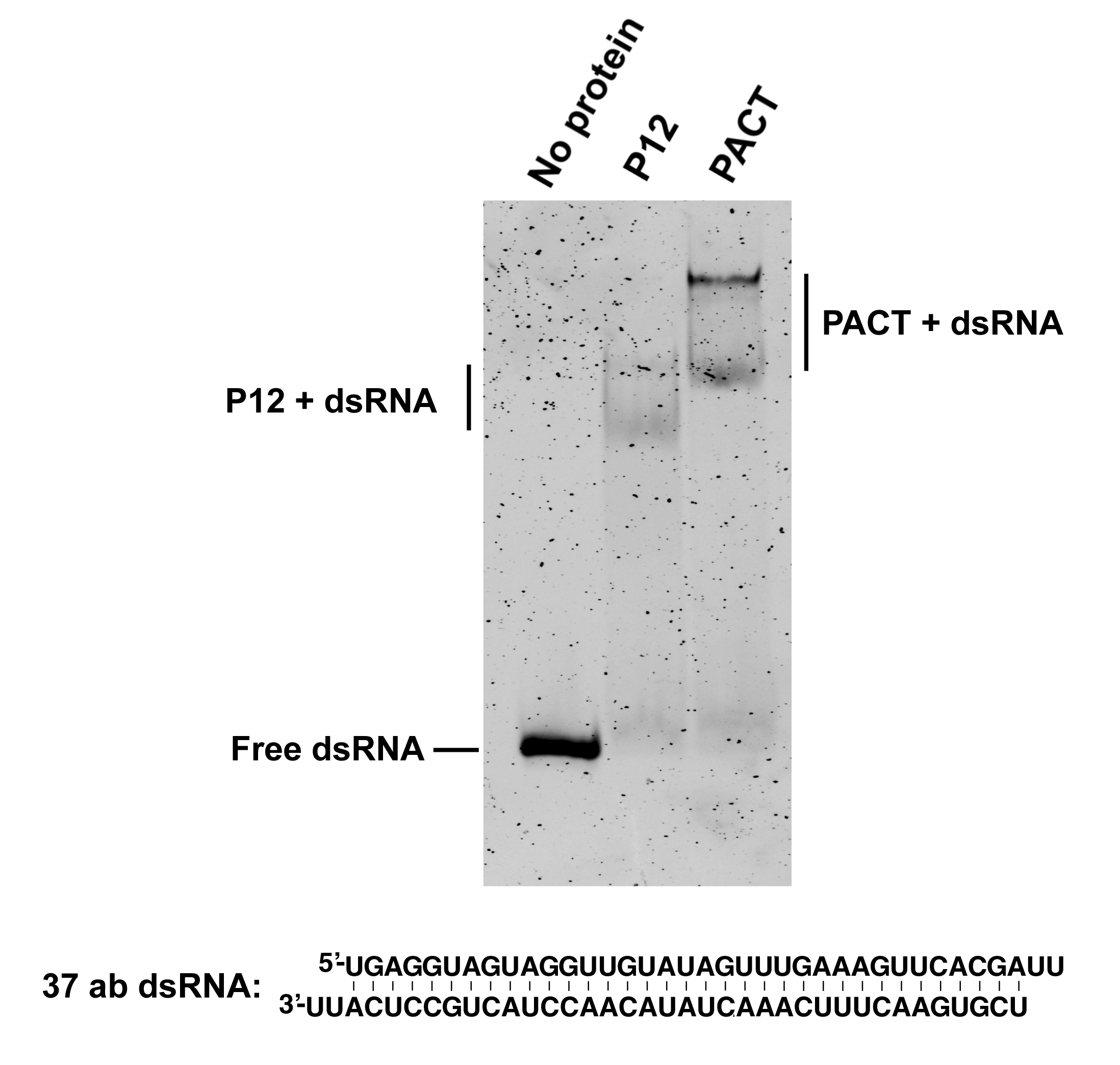
_
